# Microtubule-Associated Type II Protein Kinase A Is Important for Neurite Elongation

**DOI:** 10.1371/journal.pone.0073890

**Published:** 2013-08-13

**Authors:** Yung-An Huang, Jun-Wei Kao, Dion Tzu-Huan Tseng, Wen-Shin Chen, Ming-Han Chiang, Eric Hwang

**Affiliations:** 1 Institute of Bioinformatics and Systems Biology, National Chiao Tung University, Hsinchu, Taiwan; 2 Department of Biological Science and Technology, National Chiao Tung University, Hsinchu, Taiwan; 3 Institute of Molecular Medicine and Bioengineering, National Chiao Tung University, Hsinchu, Taiwan; 4 Center for Bioinformatics Research, National Chiao Tung University, Hsinchu, Taiwan; University of Fukui, Faculty of Medical Sciences, Japan

## Abstract

Neuritogenesis is a process through which neurons generate their widespread axon and dendrites. The microtubule cytoskeleton plays crucial roles throughout neuritogenesis. Our previous study indicated that the amount of type II protein kinase A (PKA) on microtubules significantly increased upon neuronal differentiation and neuritogenesis. While the overall pool of PKA has been shown to participate in various neuronal processes, the function of microtubule-associated PKA during neuritogenesis remains largely unknown. First, we showed that PKA localized to microtubule-based region in different neurons. Since PKA is essential for various cellular functions, globally inhibiting PKA activity will causes a wide variety of phenotypes in neurons. To examine the function of microtubule-associated PKA without changing the total PKA level, we utilized the neuron-specific PKA anchoring protein MAP2. Overexpressing the dominant negative MAP2 construct that binds to type II PKA but cannot bind to the microtubule cytoskeleton in dissociated hippocampal neurons removed PKA from microtubules and resulted in compromised neurite elongation. In addition, we demonstrated that the association of PKA with microtubules can also enhance cell protrusion using the non-neuronal P19 cells. Overexpressing a MAP2 deletion construct which does not target PKA to the microtubule cytoskeleton caused non-neuronal cells to generate shorter cell protrusions than control cells overexpressing wild-type MAP2 that anchors PKA to microtubules. Finally, we demonstrated that the ability of microtubule-associated PKA to promote protrusion elongation was independent of MAP2 phosphorylation. This suggests other proteins in close proximity to the microtubule cytoskeleton are involved in this process.

## Introduction

Protein kinase A (PKA) is a heterotetrameric enzyme consists of two regulatory and two catalytic subunits [1,2]. Four regulatory subunit isoforms (RIα, RIβ, RIIα, RIIβ) have been identified, and they maintain the holoenzyme in an inactive state. Upon cooperative binding of cAMP molecules, catalytic subunits are released from the regulatory subunits and become activated. PKA isoforms are classified into types I and II based on the regulatory subunits, and type II PKA has been shown to be the predominant form in neurons [3].

Specificity of PKA signaling is attributed to the compartmentalization of PKA [4,5]. The subcellular localization of PKA, especially type II PKA, is controlled by A kinase anchoring proteins (AKAPs). AKAPs typically contain a PKA-binding domain which binds to the regulatory subunits of PKA and a second domain that binds to the cytoskeleton or intracellular scaffolds, thus targeting PKA to specific subcellular locations [6]. It has been shown that microtubule-associated protein 2 (MAP2) is the predominant AKAP in neurons, MAP2 deletion significantly reduced various PKA subunits in dendrites [7].

PKA plays numerous important roles in neurons due to its broad range of substrates regulating neuronal processes. For instance, netrin-induced growth cone filopodia formation was dependent upon Ena/VASP phosphorylation by PKA [8]. Phosphorylation of synapsin by PKA resulted in the enhancement of neurite outgrowth [9]. In addition, PKA/cAMP signaling pathway triggers axon differentiation via phosphorylation of LKB and GSK-3β [10–12]. PKA also phosphorylates stargazin to regulate the trafficking of AMPA receptors and plays prominent roles in synaptic plasticity [13–15]. Furthermore, PKA phosphorylates NDE1 and alters its interaction with NDEL1 and LIS1 [16,17]. These proteins play cooperative and critical roles in neuronal proliferation, differentiation, and migration within the brain [18–20].

We have previously detected a significant increase of type II PKA on microtubules upon neuronal differentiation and neuritogenesis (Hwang et al., unpublished data). This increase is most likely due to the presence of MAP2 on neuronal microtubules. Despite various researches on PKA, the importance of microtubule associated PKA remains elusive. Here we used several methods to specifically reduce or enhance the amount of PKA on microtubules without altering the overall PKA level. Our data indicate that microtubule-associated PKA is important for the elongation of cellular protrusions and neurites. Furthermore, the neurite enhancing effect of PKA is independent of MAP2 phosphorylation. This result suggests other PKA substrates bound to or in close proximity to the microtubules are involved.

## Results

### PKA localizes to the microtubule cytoskeleton in neurons

**Figure 1:**
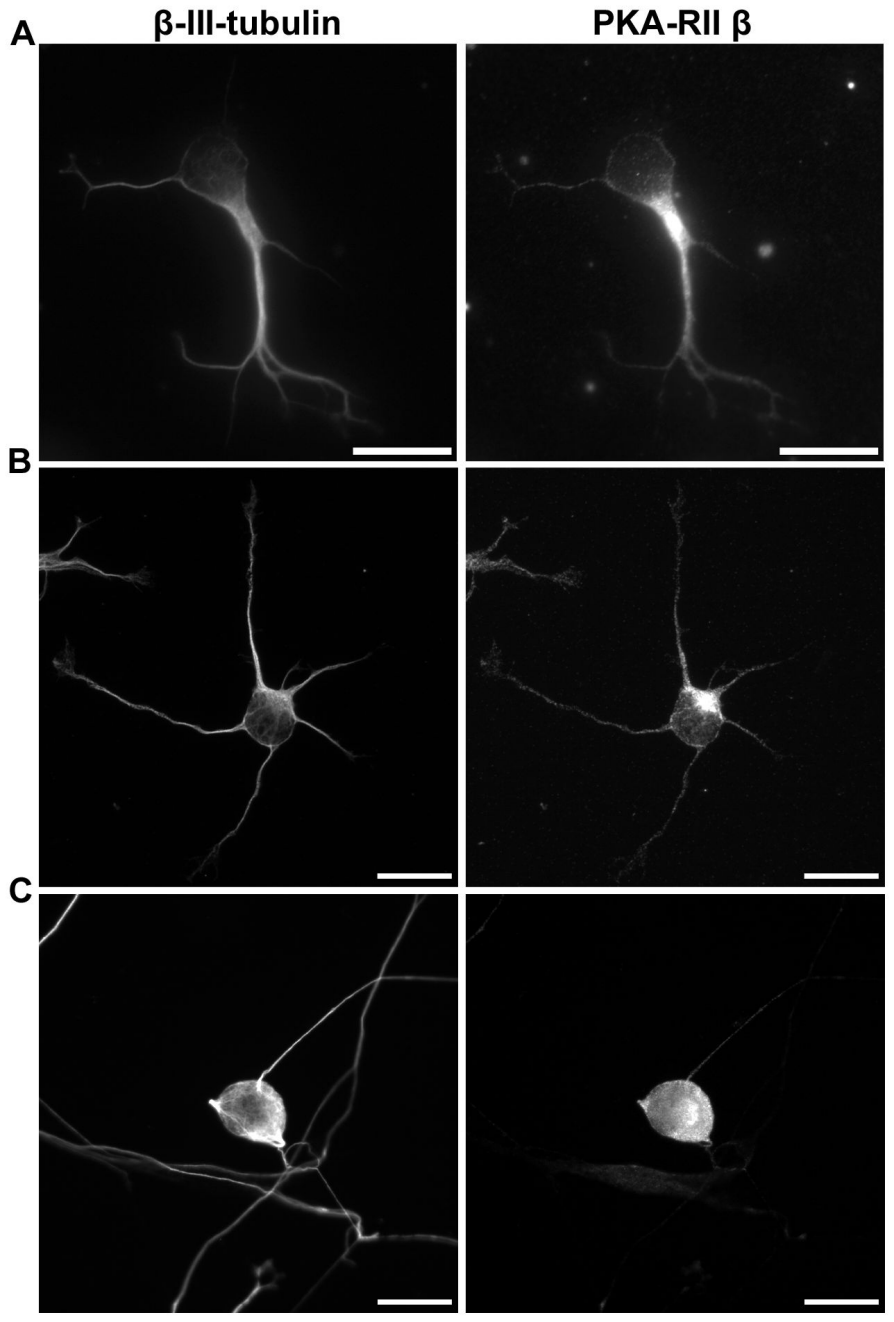
PKA-RIIβ localized to microtubule-rich regions in neurons. Immunofluorescence staining of microtubules (left) and PKA-RIIβ (right) in (A) retinoic acid-induced P19 neurons fixed at 1 day post-dissociation, (B) dissociated primary hippocampal neurons fixed at 1DIV, (C) dissociated primary dorsal root ganglion neurons fixed at 2DIV. All scale bars represent 20 µm.

Our previous proteomic study with mouse embryonal carcinoma cells (P19 cells) discovered that the amount of type II PKA complex subunits on microtubules increased upon neuronal differentiation and neuritogenesis (Hwang et al., unpublished data). This increase of microtubule-associated PKA can be detected shortly after differentiated P19 neurons were plated, suggesting that PKA-microtubule interaction may be required during early neuritogenesis. To demonstrate that PKA does localize to the microtubule cytoskeleton in young neurons, we examined the localization of PKA regulatory subunit IIβ in three different neuronal cultures. PKA regulatory subunit IIβ was selected because it is the most abundant PKA subunit in neurons [21]. Embryonal carcinoma P19 differentiated neurons, dissociated hippocampal neurons, and dissociated dorsal root ganglion (DRG) neurons were examined. When one day post-dissociation P19 neurons were stained with antibody against PKA-RIIβ and the neuron-specific β-III-tubulin, PKA-RIIβ subunits were readily detected in microtubule-based structures such as the neurite shaft (Figure 1A). Similar PKA-RIIβ localization was observed in dissociated hippocampal neurons (Figure 1B). On the other hand, PKA-RIIβ subunits were primarily detected in the soma and the proximal portion of neurites in DRG neurons (Figure 1C). Strong MAP2 staining was also detected in soma and the proximal portions of neurites in DRG neurons (Figure S1). This MAP2 localization in DRG neurons is consistent with previous observation [22] and correlates with the PKA-RIIβ localization. These observations indicate that PKA does localize to the microtubule cytoskeleton in neurons and this localization depends on the presence of the MAP2 protein.

### Interfering with the Association of Microtubules and PKA Disrupts Neurite Elongation in Hippocampal Neurons

**Figure 2:**
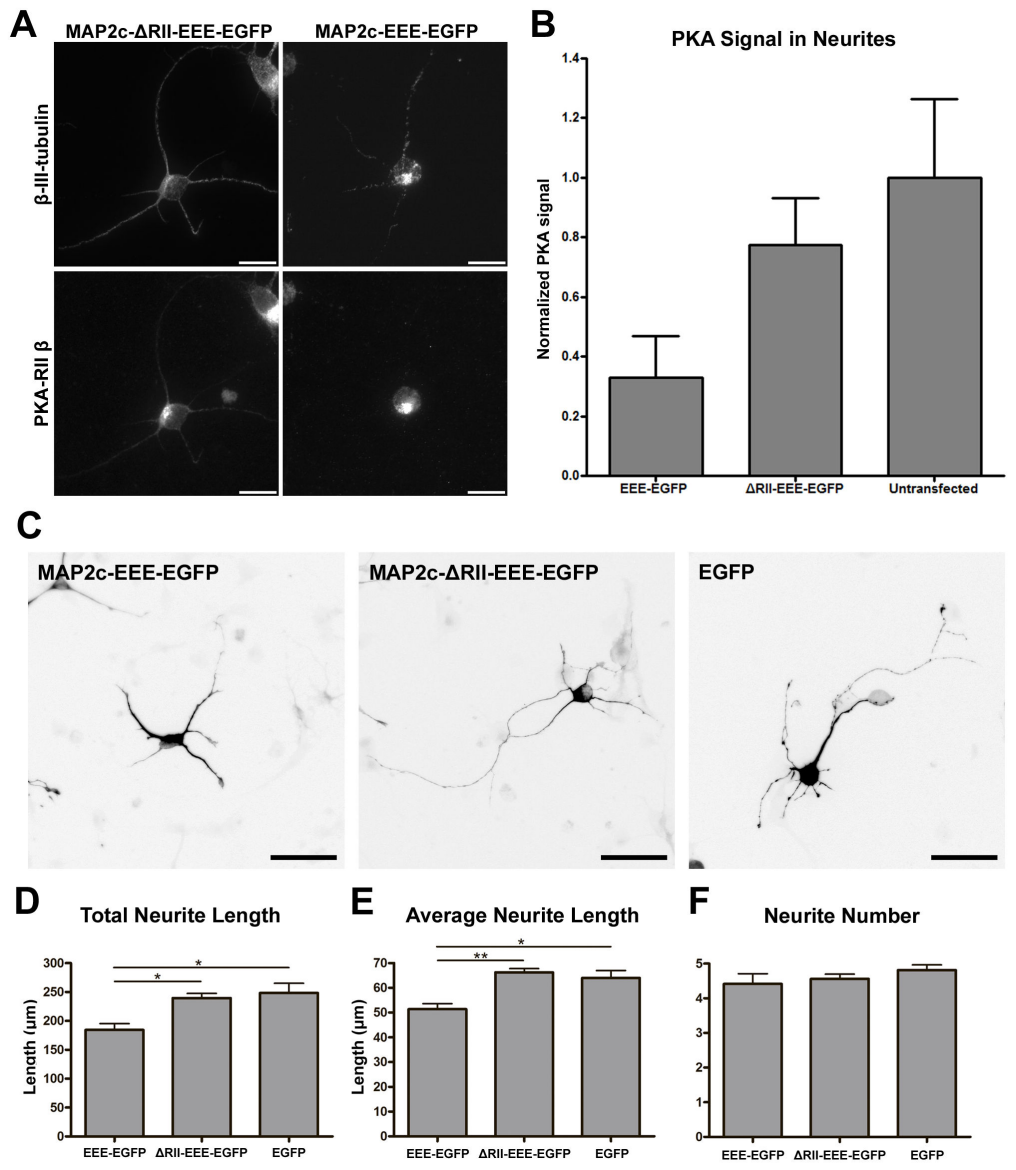
Removing PKA from microtubules using MAP2c constructs resulted in shorter neurites in dissociated hippocampal neurons. (A) E18 mouse hippocampal neurons were transfected with plasmid overexpressing MAP2c-ΔRII-EEE-EGFP (left column) or MAP2c-EEE-EGFP (right column) immediately after dissociation, incubated for 48 hours, and permeabilized with triton X-100 before formaldehyde fixation. Fixed neurons were stained with antibody against β-III-tubulin (top column) and antibody against PKA-RIIβ subunit (bottom). All scale bars equal 20 µm. (B) Quantification of microtubule-associated PKA in neurites. Error bars represent SD. All PKA signals were normalized to the PKA signal in untransfected neurons. (C) E18 mouse hippocampal neurons were transfected with plasmid overexpressing MAP2c-EEE-EGFP, MAP2c-ΔRII-EEE-EGFP, or EGFP immediately after dissociation and fixed after 48 hours. All images show signal from EGFP and were inverted to improve the visual presentation. All scale bars represent 50 µm. Quantification of total neurite length per neuron (D), average neurite length (E), and average neurite number per neuron (F) in transfected 2DIV hippocampal neurons. * p < 0.05, ** p < 0.01, one way ANOVA followed by Tukey’s post-hoc analysis. Error bars represent SEM from 3 independent repeats. More than 190 neurons were analyzed for each constructs.

To determine whether microtubule-associated PKA is important for early neuritogenesis, we utilized two MAP2c mutant constructs to alter the association of type II PKA and microtubules without changing the overall PKA level in dissociated mouse hippocampal neurons. The MAP2c-EEE-EGFP construct (Figure S2), in which all three PKA phosphorylation sites were mutated to glutamic acid to mimic constitutive phosphorylation, has previously been shown to lack the ability to localize on the microtubule cytoskeleton [23]. The MAP2c-ΔRII-EEE-EGFP construct (Figure S2), in which the PKA-RII-binding domain was removed in addition to the triple glutamic acid mutation, cannot bind to the microtubule cytoskeleton or type II PKA [24]. The MAP2c-EEE-EGFP construct serves as a dominant negative molecule that binds to and alters the localization of type II PKA, while MAP2c-ΔRII-EEE-EGFP lacks the PKA-RII-binding domain and hence does not alter type II PKA localization. To determine whether these MAP2c constructs can influence the localization of PKA on microtubules, mouse hippocampal neurons were transfected with MAP2c-EEE-EGFP or MAP2c-ΔRII-EEE-EGFP constructs immediately after dissociation and cultured for 48 hours. Transfected cells were permeabilized with triton X-100 before fixation to remove the cytoplasmic PKA and to reveal the microtubule-associated PKA. We will refer to this process of permeabilization before fixation as “cytosolic extraction” from now on. We detected substantially less PKA-RIIβ on microtubules inside neurites in dissociated hippocampal neurons overexpressing the MAP2c-EEE-EGFP construct than those overexpressing the MAP2c-ΔRII-EEE-EGFP construct (Figure 2A and 2B). In addition, the β-III-tubulin staining in these MAP2c-EEE-EGFP overexpressing neurons showed severely fragmented pattern after cytosolic extraction (Figure 2A). This kind of fragmented staining pattern was not detectable if neurons were fixed directly without cytosolic extraction. On the other hand, the staining of β-III-tubulin in MAP2c-ΔRII-EEE-EGFP overexpressing neurons appeared much less fragmented. This result implies that removing PKA from microtubules may compromise microtubule stability. When neurite morphology of hippocampal neurons overexpressing these MAP2c mutant constructs was analyzed at 2 days *in vitro* (2DIV), neurons overexpressing MAP2c-EEE-EGFP possessed significantly shorter neurites than those overexpressing MAP2c-ΔRII-EEE-EGFP or EGFP alone (Figure 2C ~ 2E). Interestingly, no significant difference in the number of neurites was detected in neurons overexpressing these constructs (Figure 2F). In addition, we did not observe significant difference in neurite branching pattern. This did not come as a surprise given that neurites barely branch at this early stage. These data suggested that the association between type II PKA and the microtubule cytoskeleton is important for neurite elongation but not for neurite initiation in dissociated hippocampal neurons.

**Figure 3:**
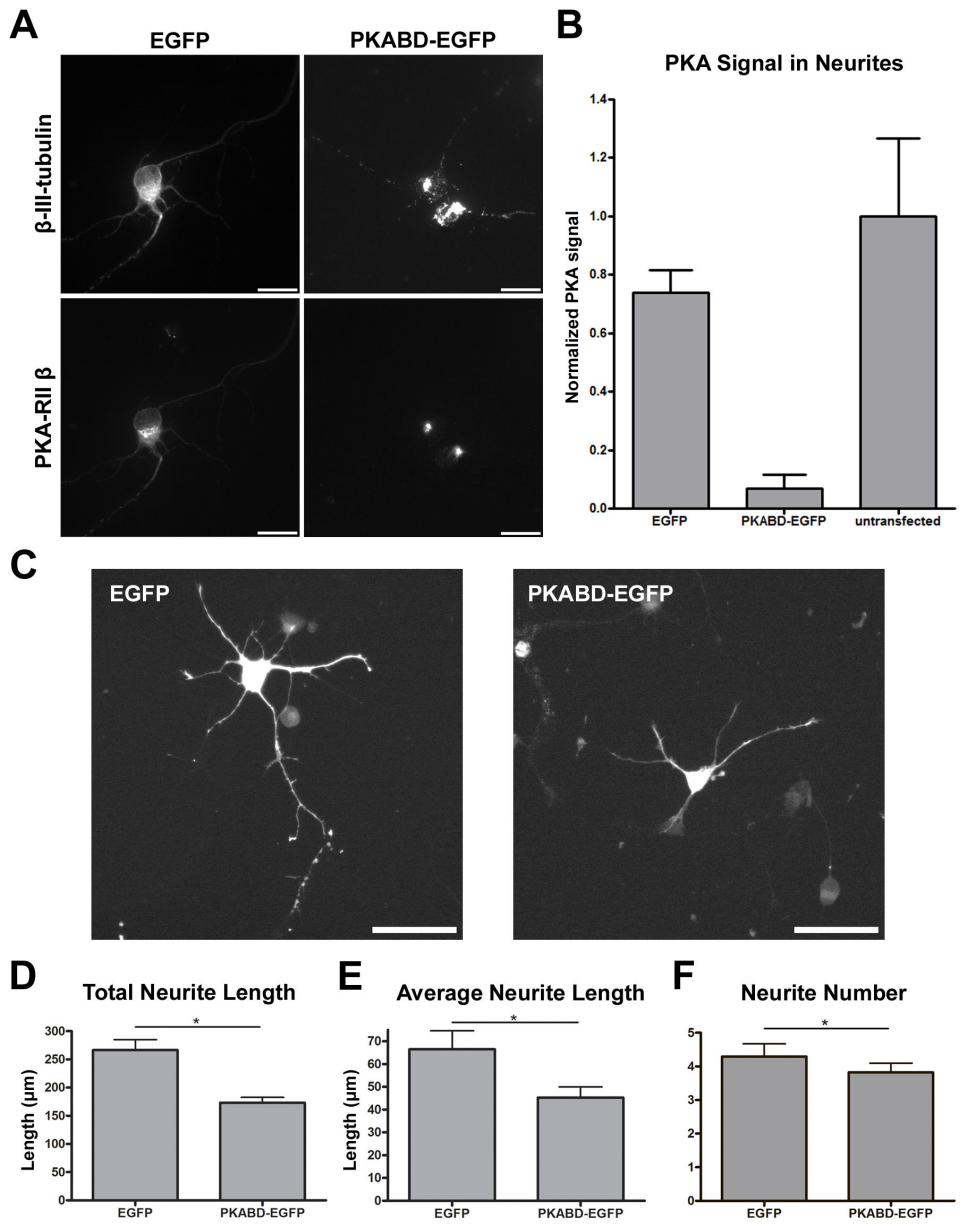
MAP2c-independent removal of PKA from microtubules resulted in shorter neurites in dissociated hippocampal neurons. (A) E18 mouse hippocampal neurons were transfected with plasmid overexpressing EGFP (left column) or PKABD-EGFP (right column) immediately after dissociation, incubated for 48 hours, and permeabilized with triton X-100 before formaldehyde fixation. Fixed neurons were stained with antibody against β-III-tubulin (top column) and antibody against PKA-RIIβ subunit (bottom). All scale bars represent 20 µm. (B) Quantification of microtubule-associated PKA in neurites. Error bars represent SD. All PKA signals were normalized to the PKA signal in untransfected neurons. (C) E18 mouse hippocampal neurons were transfected with plasmid overexpressing EGFP or PKABD-EGFP immediately after dissociation and fixed after 48 hours. All images show signal from EGFP and all scale bars represent 50 µm. Quantification of total neurite length per neuron (D), average neurite length (E), and average neurite number per neuron (F) in transfected 2DIV hippocampal neurons. * p < 0.05, two-tailed Student’s t-test. Error bars represent SEM from 3 independent repeats. More than 190 neurons were analyzed for each constructs.

Because MAP2c constructs were used to manipulate the localization of type II PKA, it is a possibility that the neurite elongation defect was caused by the altered MAP2c function (i.e. microtubule bundling) instead of the altered PKA localization. To eliminate this possibility, we created a construct (PKABD-EGFP) which combines the PKA-binding domain of MAP2c (amino acid 1~116) with EGFP. The PKABD-EGFP fusion protein competes with endogenous MAP2 for PKA binding and results in the removal of PKA from neurites in hippocampal neurons (Figure 3A and 3B). Consistent with the previous result, the staining of β-III-tubulin in PKABD-EGFP overexpressing neurons also appeared highly fragmented (Figure 3A). However, PKABD-EGFP does not contain the microtubule-binding domain or the actin-binding domain [25]; it therefore does not interfere with the function of endogenous MAP2c in microtubule stabilization or actin binding. When neurite morphology of hippocampal neurons overexpressing PKABD-EGFP or EGFP were examined at 2DIV, neurons overexpressing PKABD-EGFP generated significantly shorter neurites than those overexpressing EGFP alone (Figure 3C ~ 3E). We also observed a statistically significant reduction in the number of neurites in neurons overexpressing PKABD-EGFP (Figure 3F). This additional phenotype appears to come from PKABD-EGFP overexpression causing changes in PKA localization beyond the neurite microtubules (see discussion). In summary, our results demonstrate that interfering with the association of microtubules and type II PKA disrupts neurite elongation in hippocampal neurons.

### MAP2c-dependent PKA localization on microtubules can enhance neurite-like protrusion in non-neuronal cells

**Figure 4:**
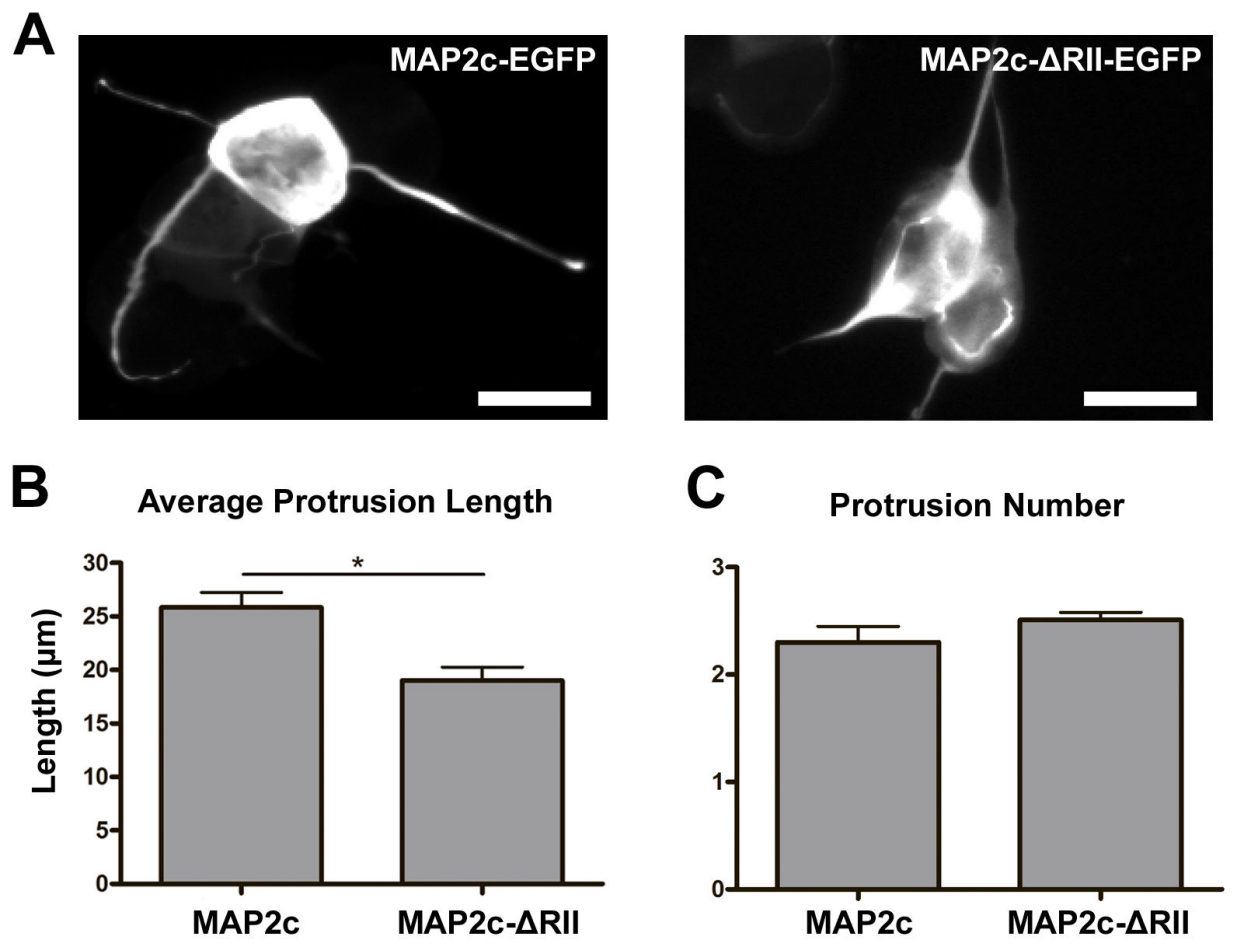
Recruiting PKA to MAP2c-stabilized microtubules resulted in longer neurite-like cell protrusions in non-neuronal cells. (A) Images of non-neuronal P19 cells transfected with plasmid overexpressing MAP2c-EGFP (left) or MAP2c-ΔRII-EGFP (right) for 20 hours, incubated with 1 µM of latrunculin-A for 4 hours, and fixed with formaldehyde. All images show signal from EGFP and all scale bars represent 20 µm. Quantification of average protrusion length (B) and average protrusion number per cell (C). * p < 0.05, two-tailed Student’s t-test. Error bars represent SEM from three independent repeats. More than 300 cells were analyzed for each constructs.

Our previous loss-of -function experiments showed that removing PKA from microtubules disrupts proper neurite elongation. In order to demonstrate that the association between microtubule and PKA is indeed important for cellular elongation, we wanted to examine whether allocating more PKA onto microtubules can enhance elongation (i.e. gain-of-function experiments). To test this, we utilized the undifferentiated P19 cells that do not express detectable MAP2 protein [26]. This system allowed us to examine the function of microtubule-associated PKA without the interference of endogenous MAP2c. We used two MAP2c constructs to alter the association of PKA and the microtubule cytoskeleton: the wild type MAP2c fused to EGFP (MAP2c-EGFP) and the MAP2c protein lacking the PKA-RII-binding domain fused to EGFP (MAP2c-ΔRII-EGFP) (Figure S2) [24]. The MAP2c-EGFP construct binds to and readily targets PKA onto microtubules whereas the MAP2c-ΔRII-EGFP construct binds to but cannot direct PKA onto microtubules (Figure S3). Previous study showed that non-neuronal cells can be induced to generate neurite-like cell protrusions when treated with a microtubule stabilizing reagent (e.g. MAP2 overexpression or taxol) and an F-actin destabilizing drug (e.g. latrunculin or cytochalasin) [24,27]. A similar neurite-like cell protrusions can also be generated by treating MAP2c-EGFP- or MAP2c-ΔRII-EGFP-overexpressing cells with latrunculin-A (Lat-A). When undifferentiated P19 cells overexpressing MAP2c-EGFP or MAP2c-ΔRII-EGFP were exposed to 1 µM of Lat-A, neurite-like cell protrusions can be easily observed after 4 hours. The length of the cell protrusions in P19 cells overexpressing MAP2c-EGFP was significantly longer than cells overexpressing MAP2c-ΔRII-EGFP (Figure 4A and 4B). However, the number of neurite-like protrusion per cell did not show significant difference between cells overexpressing MAP2c-EGFP and MAP2c-ΔRII-EGFP (Figure 4C). These results correlate with our previous observation in neurons and indicate that PKA-microtubule association can enhance the elongation but not the formation of cell protrusions.

### The protrusion enhancing ability of microtubule-associated PKA is independent of MAP2c phosphorylation

**Figure 5:**
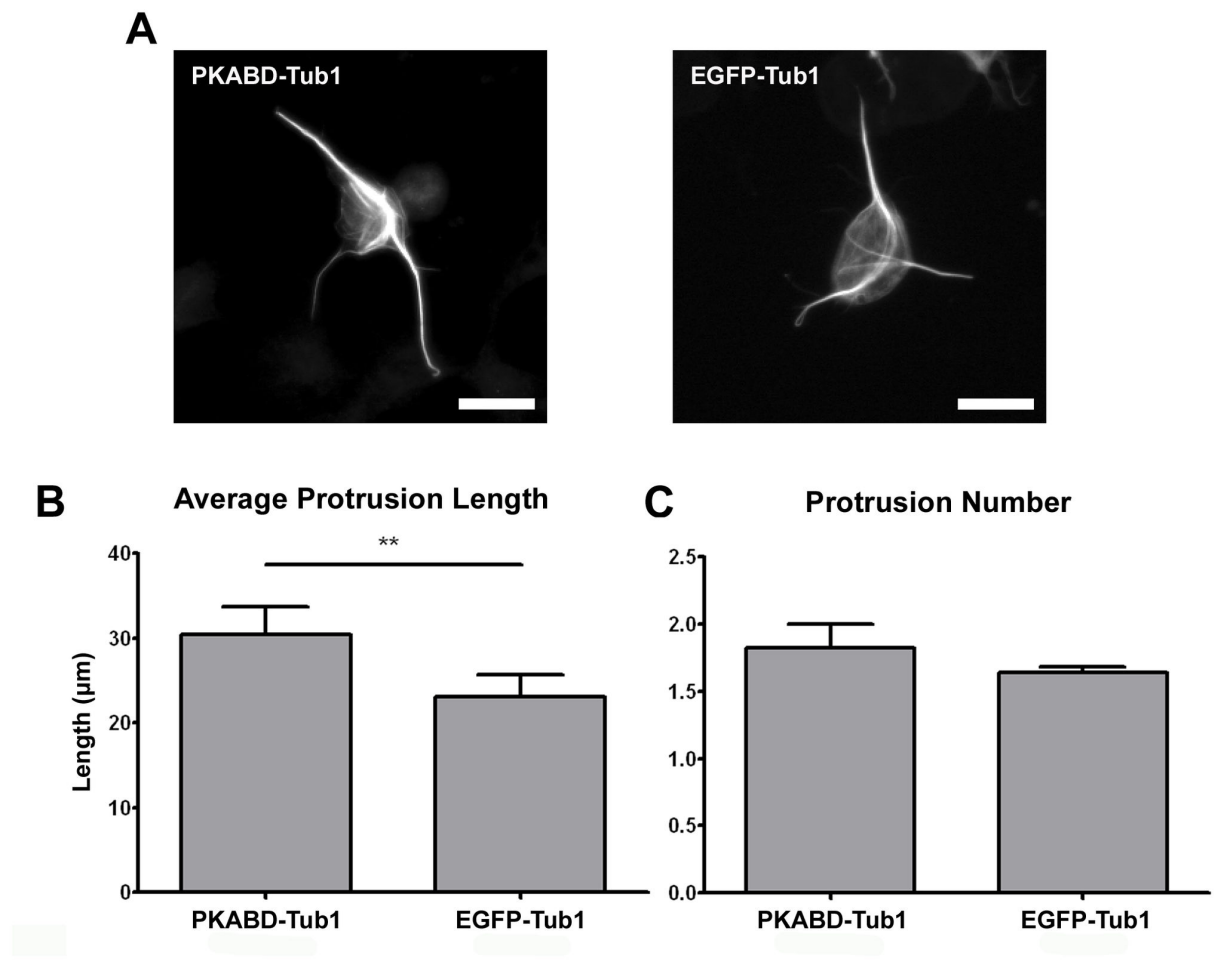
MAP2c-independent PKA recruitment onto microtubules resulted in longer neurite-like cell protrusions in non-neuronal cells. (A) Image of P19 cells transfected with plasmid overexpressing PKABD-Tub1 (left) or EGFP-Tub1 (right) constructs for 20 hours, incubated with 1 µM of latrunculin-A and 0.5 µM of taxol for 4 hours, and formaldehyde fixed. All scale bars represent 20 µm. Quantification of average protrusion length (B) or average protrusion number per cell (C). ** p < 0.01, two-tailed Student’s t-test. Error bars represent SEM from three independent repeats. More than 120 cells were analyzed for each constructs.

One of the major substrates for PKA in close proximity to microtubules is the MAP2 protein, and it has been shown that MAP2 phosphorylation switches its localization from microtubules towards actin filaments [23]. Therefore, it is possible that the cell protrusion promoting effect of microtubule-associated PKA depends solely on the phosphorylation of MAP2c. To test this possibility, we examined the protrusion enhancing ability of microtubule-associated PKA in the absence of MAP2c phosphorylation domain. A PKABD-Tub1 construct combines the PKA-binding domain of MAP2c (amino acid 1~116) with the human α-tubulin was used for this study. This PKABD-Tub1 construct allows us to recruit endogenous type II PKA onto microtubules in undifferentiated P19 cells in the absence of PKA phosphorylation sites on MAP2. To induce the formation of neurite-like protrusions in undifferentiated P19 cells, we applied 0.5 µM of taxol and 1 µM of Lat-A to cells for 4 hours. PKA-RIIβ can be observed to colocalize with the microtubule cytoskeleton in cells overexpressing PKABD-Tub1 but not in those overexpressing EGFP-Tub1 (Figure S4). P19 cells overexpressing PKABD-Tub1 possessed longer cell protrusions than control cells overexpressing EGFP-Tub1 (Figure 5A and 5B). However, there was no statistically significant difference in protrusion number between cells overexpressing PKABD-Tub1 and cells overexpressing EGFP-Tub1 (Figure 5C). To exclude the possibility that PKABD-Tub1 and EGFP-Tub1 may influence the microtubule stability differently and hence resulted in different cell protrusion length, we analyzed the stability of the microtubule cytoskeleton in cells overexpressing PKABD-Tub1 or EGFP-Tub1. We used 2 different approaches to quantify the effect of these constructs on microtubule stability. First, we treated P19 cells overexpressing PKABD-Tub1 or EGFP-Tub1 with various dosage of microtubule-destabilizing drug nocodazole and quantified the length of the entire microtubule cytoskeleton (Figures S5A and S5C). We reasoned that if PKABD-Tub1 and EGFP-Tub1 do influence microtubule stability, we should be able to detect the difference in microtubule length at a specific nocodazole concentration. Second, we quantified the amount of acetylated microtubules in P19 cells overexpressing PKABD-Tub1 or EGFP-Tub1 (Figures S5B and S5D). It has been shown that stable microtubules accumulated more α-tubulin acetylation modifications. Neither analyses revealed any difference in microtubule stability in cells overexpressing PKABD-Tub1 or EGFP-Tub1. In summary, our result indicates that PKA’s ability to enhance the elongation of cell protrusion is independent of MAP2c phosphorylation and suggests that other microtubule-associated or microtubule-proximal molecules may be involved.

## Discussion

In this study, we demonstrated that the microtubule-associated PKA is involved in neurite elongation and cell protrusion elongation. We observed a MAP2-depndent localization of PKA on microtubules, PKA can be detected along the entire length of the neurite in neurons with high level of MAP2 (e.g. hippocampal neurons and P19 neurons) while PKA was primarily detected in proximal neurites in neurons with low level of MAP2 (DRG neurons) (Figures 1 and S1). This MAP2-dependent PKA localization may provide an explanation for the discrepancy in the localization of type II PKA in mouse hippocampal neurons (PKA was detected along the entire neurite shaft) and *Xenopus* spinal neurons (PKA was only detected at the growth cone) [5,28].

We observed a decrease of neurite length in dissociated hippocampal neurons when the amount of microtubule-associated PKA was reduced using a MAP2c construct that is unable to associate with the microtubule (Figure 2), or using a PKA-binding domain-EGFP fusion construct (Figure 3). Interestingly, removing PKA from microtubules by overexpressing MAP2c-EEE-EGFP did not affect the number of neurites formed but overexpressing PKABD-EGFP did. One explanation came from the observation that PKABD-EGFP overexpression caused changes in PKA localization beyond the neurite microtubules. While PKABD-EGFP and MAP2c-EEE-EGFP overexpression both removed PKA from the microtubule inside neurites, PKABD-EGFP overexpression caused PKA to condense to a single focus inside the soma – presumably the centrosome (Figure 3A). This observation implies that PKABD-EGFP overexpression may alter PKA localization on other cellular structures, and might explain why there was a neurite initiation defect in PKABD-EGFP overexpressing neurons but not in MAP2c-EEE-EGFP overexpressing neurons. Nonetheless, these results show that the microtubule-associated PKA is important for neurite elongation. Our result is consistent with the observation that neurite length was reduced in dissociated hippocampal neurons isolated from MAP2 knockdown mice compared to the wild-type control [7]. Another study showed that a MAP2 mutant line of mice expressing the truncated form of MAP2 lacking the PKA-binding domain displayed abnormalities in hippocampal architecture, particularly in CA1 region. Dendrites of CA1 neurons are greatly disarrayed, and the molecular layer is absent or it is not as well defined as in wild type animals [29]. This observation is also consistent with our results that microtubule-associated PKA is important for promoting neurite elongation.

Even in cells with no detectable MAP2 expression (undifferentiated P19 cells), bringing type II PKA onto the microtubule cytoskeleton can also enhance cell protrusion (Figures 4 and 5). This suggests that the microtubule-associated substrate for PKA is not MAP2, and this substrate is present in both neurons and non-neuronal cells. One possible substrate that fits these criteria is the guanine nucleotide exchange factor of RhoA, GEF-H1. GEF-H1 localizes to the microtubules in both neurons and non-neuronal cells, and it showed a high degree of colocalization with MAP2 in the dendritic shaft of the neuron [30,31]. It has been documented that PKA can phosphorylate GEF-H1, and this phosphorylation suppressed the guanine nucleotide exchange activity of GEF-H1 on RhoA [32]. Since activated RhoA (i.e. GTP-bound RhoA) can mediate neurite retraction [33], PKA-mediated GEF-H1 phosphorylation can potentially encourage neurite elongation. The other potential substrate is the microtubule plus-end tracking protein CLIP-170. CLIP-170 has been shown to localize to the plus-end of microtubules and phosphorylation of CLIP-170 by PKA reduced this microtubule-associated population [34]. CLIP-170 suppression has recently been shown to affect neuronal polarization and neurite extension [35]. To determine the identity of this microtubule-associated PKA substrate, we plan on depleting GEF-H1 or CLIP-170 in Lat-A-treated P19 cells overexpressing MAP2c-EGFP or MAP2c-ΔRII-EGFP. When depleted, the responsible substrate will cause the P19 cell to generate similar protrusion length no matter it is overexpressing MAP2c-EGFP or MAP2c-ΔRII-EGFP.

In summary, our study reveals a specific function of the microtubule-associated PKA in promoting neurite elongation. The same mechanism is also utilized in generating cell protrusion. In addition, this PKA signaling pathway is not propagated through MAP2 phosphorylation.

## Materials and Methods (No Limit)

### Ethical Statement

Primary hippocampal neurons and dorsal root ganglion neurons were isolated from mouse embryos (C57BL/6). Timed pregnant mice and their embryos were sacrificed in accordance with the National Institute of Health Guide for the Care and Use of Laboratory Animals (NIH Publications No. 80-23), and that formal approval to conduct the experiments described has been obtained from the animal subjects review board of National Chiao Tung University.

### DNA and Plasmid constructs

Plasmids pMAP2c-EGFP and pMAP2c-ΔRII-EGFP were kindly provided by Dr. Shelley Halpain [24]. To overexpress MAP2 constructs in neurons, DNA sequence containing the chick β-actin promoter was cut from pCAG-EGFP-N2 plasmid and cloned into pMAP2c-EEE-EGFP or pMAP2c-ΔRII-EEE-EGFP using SnaBI/HindIII to create pCAG-MAP2c-EEE-EGFP and pCAG-MAP2c-ΔRII-EEE-EGFP, respectively. To construct pPKABD-Tub plasmid, the DNA fragment encoding MAP2c amino acids 1–116 (i.e. PKABD) was amplified using primers 5’-TTTCGCTAGCATGGCTGACGAGAGGAAAGA-3’ and 5’-AAAGATCTGTGTTGGGCCTCCTTCTC-3’ and cloned into the pEGFP-Tub plasmid (Clontech) using NheI and BglII. To construct the pPKABD-EGFP plasmid, the PKABD DNA fragment was amplified using primers 5’ –AAAAAAGCTTATGGCTGACGAGAGGAAAGA– 3’ and 5’ –GGAATTCGTGTTGGGCCTCCTTCTC– 3’ and cloned into the pCAG-EGFP-N2 plasmid by HindIII and EcoRI. The pCAG-EGFP-N2 plasmid is derived from pEGFP-N2 vector (Clontech) by inserting a chick β-actin promoter into the CMV IE promoter. All constructs were verified by sequencing.

### Cell cultures

Hippocampal neurons from E18 mouse embryos (C57BL/6) were prepared according to Chen et al. [36]. Dissociated hippocampal neurons were seeded onto poly-L-lysine-coated coverslips at the density of 3x10^4^ neurons per cm^2^. Neurons were fixed 2 days after seeding.

Dorsal root ganglion neurons from E15 mouse embryos (C57BL/6) were prepared according to Malin et al. [37]. Dissociated root ganglion neurons were seeded onto poly-L-lysine and laminin-coated coverslips at the density of 1x10^4^ neurons per cm^2^. Neurons were fixed 1 day after seeding.

P19 cells (acquired from American Type Culture Collection, Manassas, VA) were maintained in minimum essential medium (Invitrogen) supplemented with 10% fetal bovine serum (Biological Industries) and 1 mM sodium pyruvate. Retinoic acid-mediated P19 differentiation was performed according to Jones-Villeneuve et al. with slight modification [38]. Briefly, undifferentiated cells were trypsinized and grown in suspension for 4 days in differentiation medium (minimum essential medium supplemented with 2 mM glutamine, 1 mM pyruvate, 5% fetal bovine serum, and 0.5 µM retinoic acid) during which time they form aggregates. Retinoic acid-induced aggregates were dissociated by trypsin and plated at a density of 1x10^5^ cells per cm^2^ in serum-free neuron maintenance medium (minimum essential medium plus 2 mM glutamine, 1 mM pyruvate, 10% N2 supplements (Invitrogen), 0.6% glucose, and 0.1% ovalbumin) for 1 additional day before fixation.

### Transfection and drug treatment

P19 cells were transfected using the Lipofectamine 2000 (Invitrogen) with the ratio 2 µg of plasmid DNA to 1 µL of Lipofectamine and incubated for 20~24 hrs before drug treatment or fixation. In drug treatment experiments, P19 cells were incubated for 20 hrs before the culture medium was replaced with drug-containing medium (taxol or taxol plus latrunculin-A). Cells were incubated in drug-containing medium for 4 hrs before fixation. For dissociated hippocampal neurons, plasmids were introduced into neurons using Lonza basic neuron SCN nucleofector kit immediately after dissociation. 2x10^5^ dissociated neurons and 0.6 µg plasmid DNA were suspended in the 20 µL room temperature nucleofector solution. Electroporation was conducted with the nucleofector program SCN Basic Neuro Program 1 using the Nucleofector II. 80 µL of pre-warmed and pre-equilibrated RPMI 1640 medium (Invitrogen) was added to cuvette immediately after electroporation and transferred into a 1.5 mL tube for recovery. After 10 minutes of recovery in a 37^°^ C CO_2_ incubator, neurons were seeded onto PLL-coated coverslips at the aforementioned density.

### Indirect immunofluorescence staining and image acquisition

Cells or neurons were fixed with 3.7% formaldehyde in 1xPBS for 15 min at 37^°^ C, and permeabilized with 0.25% triton X-100 for 5 min at room temperature. For experiments in which the cytoplasmic pool of PKA needs to be removed before fixation (cytosolic extraction), cells or neurons were permeabilized in 37^°^ C extraction buffer (100 mM pH 6.9 PIPES, 10 mM EGTA, 5 mM magnesium chloride, 0.1% triton X-100) for 15 seconds and then fixed with 3.7% formaldehyde in 1xPBS for 15 min at 37^°^ C. After being washed three times with 1xPBS, cells were blocked for 1 hr at 37^°^ C with 10% BSA. Coverslips with cells were incubated for 1 h at 37^°^ C with primary antibodies (TUJ1, 1:2000, Covence MMS-435P; anti-PKA-RIIα antibody, 1:500, BD612242; anti-PKA-RIIβ antibody, 1:500, BD610625; DM1A, 1:1000, Sigma T6199; rabbit polyclonal anti-βIII-tubulin antibody, 1:1000, Novus Biologicals NB110-79872; rabbit polyclonal anti- MAP2 antibody, 1:1000; mouse monoclonal anti-acetylated α-tubulin antibody, 1:500, Abcam, ab24610; rabbit polyclonal anti-α-tubulin antibody, 1:500, Abcam, ab52866) diluted in 2% BSA. After washing, cells were incubated with Alexa Fluor 405-, DyLight 488-, Alexa Fluor 568-, or Alexa Fluor 680-labeled secondary antibodies (1:1000) for 1 h at 37^°^ C in the dark. Coverslips with cells were washed three times with 1xPBS and mounted with Fluoromount (Sigma) onto glass slides. Fluorescence images were acquired with an Olympus IX-71 inverted microscope equipped with a CoolLED fluorescent light source and a Hamamatsu ORCA-R2 camera. A 20× 0.75 N.A. and a 60× 1.35 N.A. plan apochromat objective lens were used to collect fluorescent images. MetaMorph software was used to automatically acquire 20 images starting in the center of the coverslip for P19 cells morphological quantification or manually acquire more than 60 mouse hippocampal neurons for morphological quantification.

### Neurite length analysis

Neurite-like protrusion length in P19 cells and neurite length in hippocampal neurons were manually traced with the ImageJ plugin, NeuronJ 1.4.1. In P19 cells, the length of the neurite-like protrusions was measured from the cell body edge to the tip of the protrusion. Any protrusion shorter than half of the cell body diameter was not recognized as a neurite-like protrusion. In hippocampal neurons, the length of the neurites was measured from the edge of the soma to the wrist of the growth cone. Unless it had a marked growth cone at the tip, any protrusion shorter than the diameter of its soma was not recognized as a neurite. Only EGFP and TUJ1-positive neurons were analyzed.

### Microtubule length analysis

Fluorescence images were binarized with ImageJ using the midgrey algorithm in the auto-local threshold plug-in, and the resulting binary images were processed with the particle remover plug-in and then skeletonized. Total pixels were then calculated using the “measure” function of ImageJ.

### Statistical analysis

All statistical analyses were performed using GraphPad Prism 4. Values are expressed as means ± standard error of the mean. Significant differences between the means were calculated with the two-tailed Student’s t-test or one-way ANOVA followed by Tukey’s post-hoc analysis.

## Supporting Information

Figure S1 **The localization of PKA-RIIβ in neurites depends on MAP2.** Immunofluorescence staining of MAP2 (left) and PKA-RIIβ (right) in (A) retinoic acid-induced P19 neurons fixed at 1 day post-dissociation, (B) dissociated primary hippocampal neurons fixed at 1DIV, (C) dissociated primary dorsal root ganglion neurons fixed at 2DIV. All scale bars represent 20 µm.
(TIF)

Figure S2 **MAP2c constructs used in this study.** (A) Domain diagrams of various MAP2c constructs and their ability to interact with PKA or microtubules (MT) used in this study. The location of the PKA-binding domain (PKABD) and the microtubule-binding domain (MTBD) are shown. Diagrams are not drawn to scale. (B) The localization of MAP2 constructs (top row) with regard to the microtubule cytoskeleton (bottom row) in undifferentiated P19 cells at 24 hours post-transfection.
(TIF)

Figure S3 **MAP2c mutant constructs alter the association of PKA and the microtubule cytoskeleton in non-neuronal cells.** Images of P19 cells transfected with plasmid overexpressing MAP2c-EGFP (left) or MAP2c-ΔRII-EGFP (right), incubated for 24 hours, and permeabilized with triton X-100 before formaldehyde fixation. Cells were visualized using EGFP signal (top) or immunofluorescence stained with antibody against PKA-RIIβ (bottom). All scale bars represent 5 µm.
(TIF)

Figure S4 **PKA can be recruited onto microtubules in non-neuronal cells by overexpressing the PKABD-Tub1 construct.** Images of P19 cells transfected with plasmid overexpressing PKABD-Tub1 (left) or EGFP-Tub1 (right) along with plasmid expressing TagBFP-histone H2B, incubated for 24 hours, and permeabilized with triton X-100 before formaldehyde fixation. Cells were immunofluorescence stained with antibody against tubulin (top row) and PKA-RIIβ (second row). TagBFP-H2B (third row) was used to identify the transfected cells. All scale bars represent 10 µm.
(TIF)

Figure S5 **PKABD-Tub1 and EGFP-Tub1 did not affect the stability of the microtubule cytoskeleton.** (A) Fluorescent images of untransfected P19 cells (top), P19 cells cotransfected with TagBFP-histone H2B and EGFP-Tub1 (middle), or P19 cells cotransfected with TagBFP-histone H2B and PKABD-Tub1 (bottom) for 23 hours, incubated with 0.2% DMSO (control) or different concentration of nocodazole for 1 hours, and formaldehyde fixed. The α-tubulin staining is shown in red, DAPI staining (top) or TagBFP (middle and bottom) are shown in blue. (B) Fluorescent images of acetylated-microtubule (Ac-MT) in untransfected P19 cells (left), P19 cells transfected with EGFP-Tub1 (middle), or P19 cells transfected with PKABD-Tub1 (right) and fixed at 24 hours. All scale bars represent 20 µm. (C) Quantification of total microtubule length in P19 cells from panel A. (D) Quantification of the ratio of acetylated-microtubules to total microtubules in P19 cells from panel B. All error bars represent SD. More than 15 cells were analyzed per condition per construct.
(TIF)
