# Supplementary figures and images for: Microtubule-Associated Type II Protein Kinase A Is Important for Neurite Elongation
Source: PLoS One. 2013 Aug 13;8(8):e73890. doi: 10.1371/journal.pone.0073890 (PMC3742546; doi:10.1371/journal.pone.0073890)

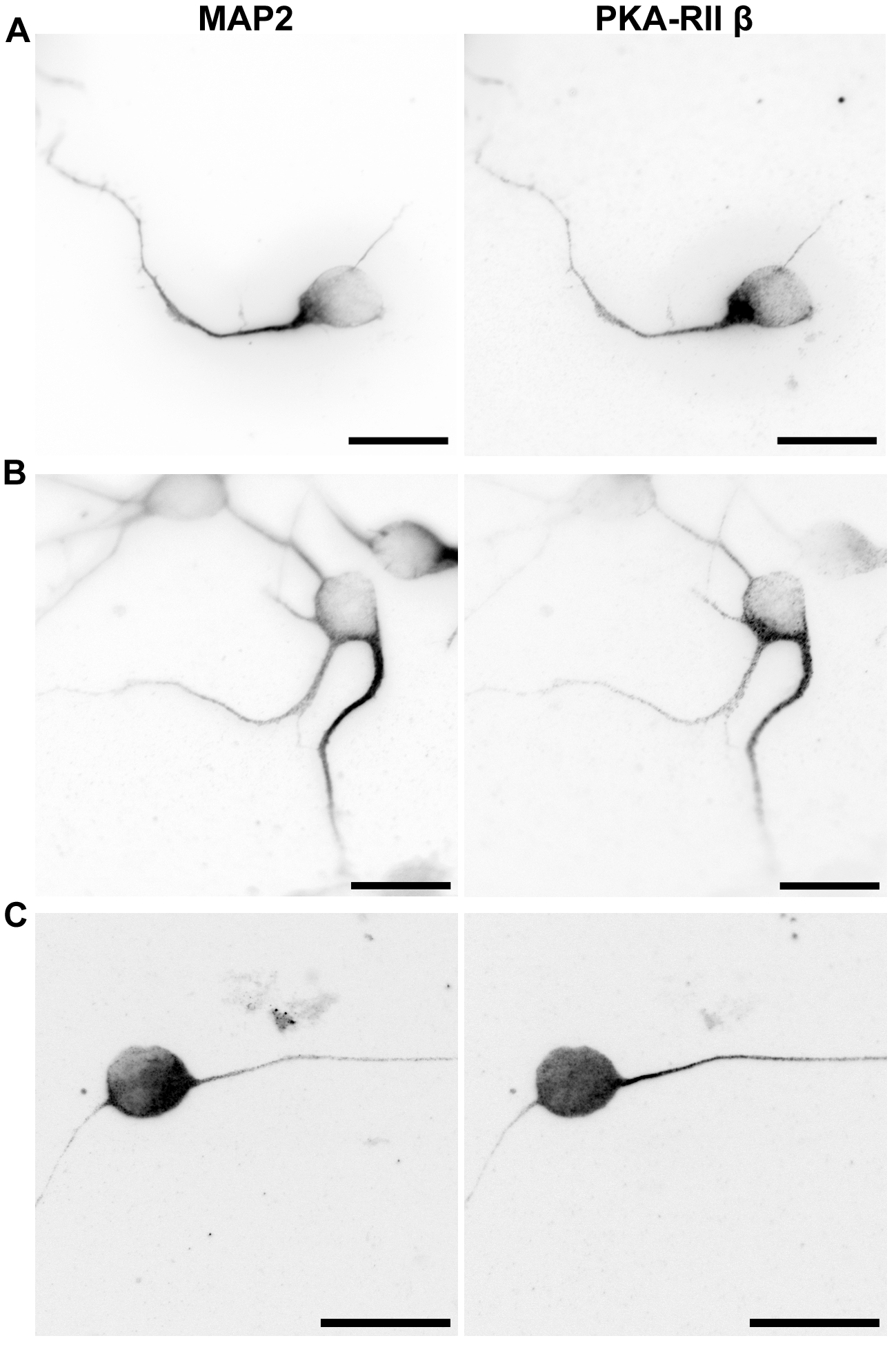

Supplement: Figure S1 — The localization of PKA-RIIβ in neurites depends on MAP2. Immunofluorescence staining of MAP2 (left) and PKA-RIIβ (right) in (A) retinoic acid-induced P19 neurons fixed at 1 day post-dissociation, (B) dissociated primary hippocampal neurons fixed at 1DIV, (C) dissociated primary dorsal root ganglion neurons fixed at 2DIV. All scale bars represent 20 µm. (TIF) [file pone.0073890.s001.tif]

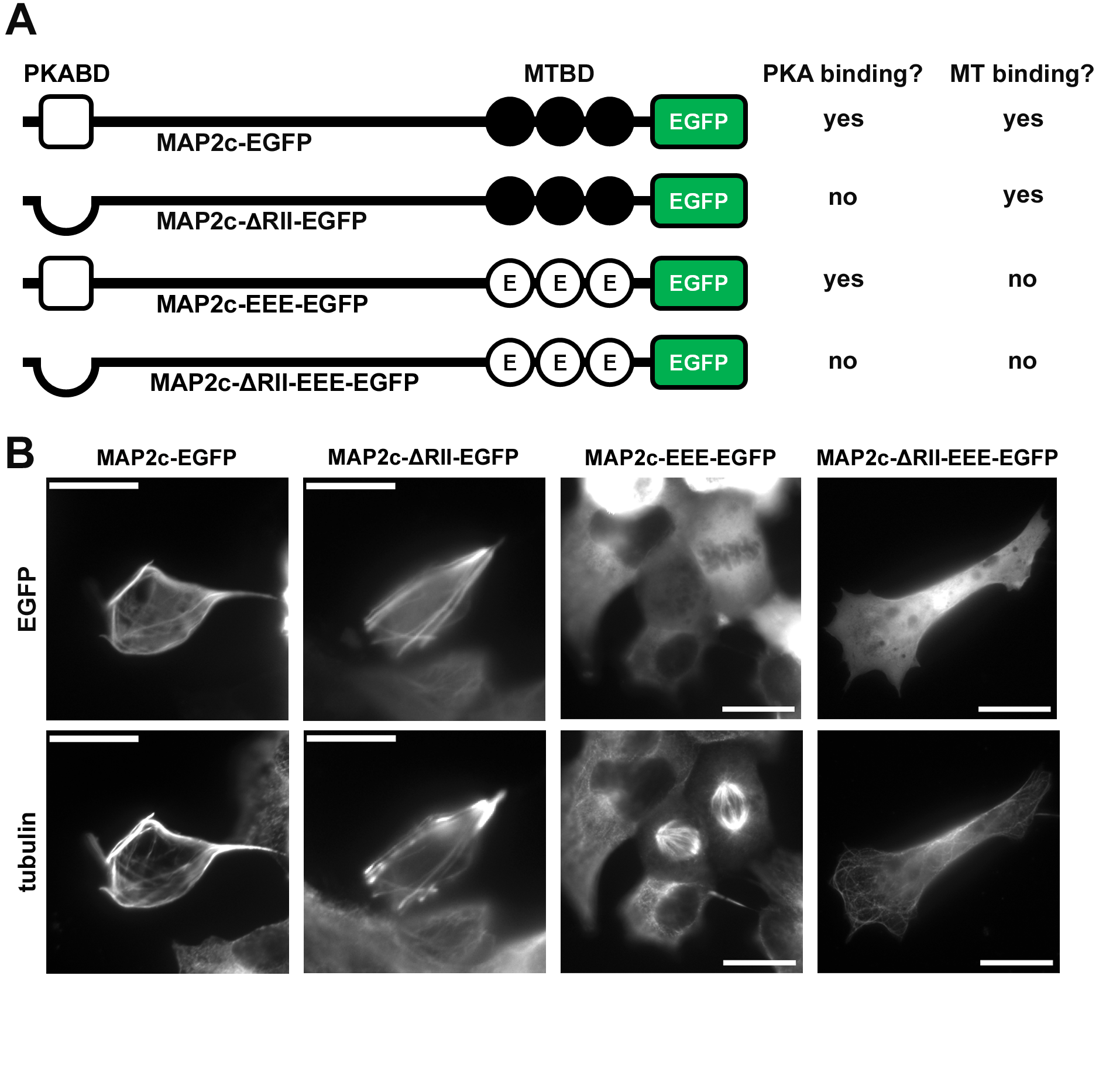

Supplement: Figure S2 — MAP2c constructs used in this study. (A) Domain diagrams of various MAP2c constructs and their ability to interact with PKA or microtubules (MT) used in this study. The location of the PKA-binding domain (PKABD) and the microtubule-binding domain (MTBD) are shown. Diagrams are not drawn to scale. (B) The localization of MAP2 constructs (top row) with regard to the microtubule cytoskeleton (bottom row) in undifferentiated P19 cells at 24 hours post-transfection. (TIF) [file pone.0073890.s002.tif]

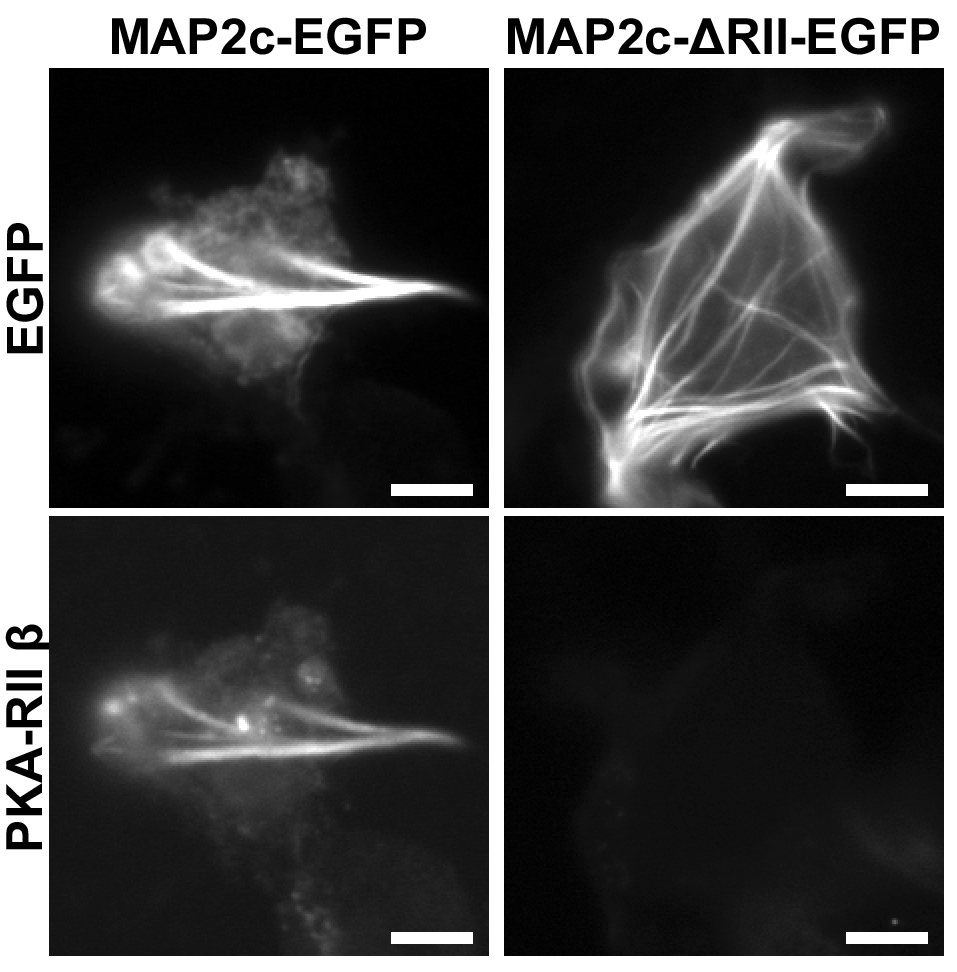

Supplement: Figure S3 — MAP2c mutant constructs alter the association of PKA and the microtubule cytoskeleton in non-neuronal cells. Images of P19 cells transfected with plasmid overexpressing MAP2c-EGFP (left) or MAP2c-ΔRII-EGFP (right), incubated for 24 hours, and permeabilized with triton X-100 before formaldehyde fixation. Cells were visualized using EGFP signal (top) or immunofluorescence stained with antibody against PKA-RIIβ (bottom). All scale bars represent 5 µm. (TIF) [file pone.0073890.s003.tif]

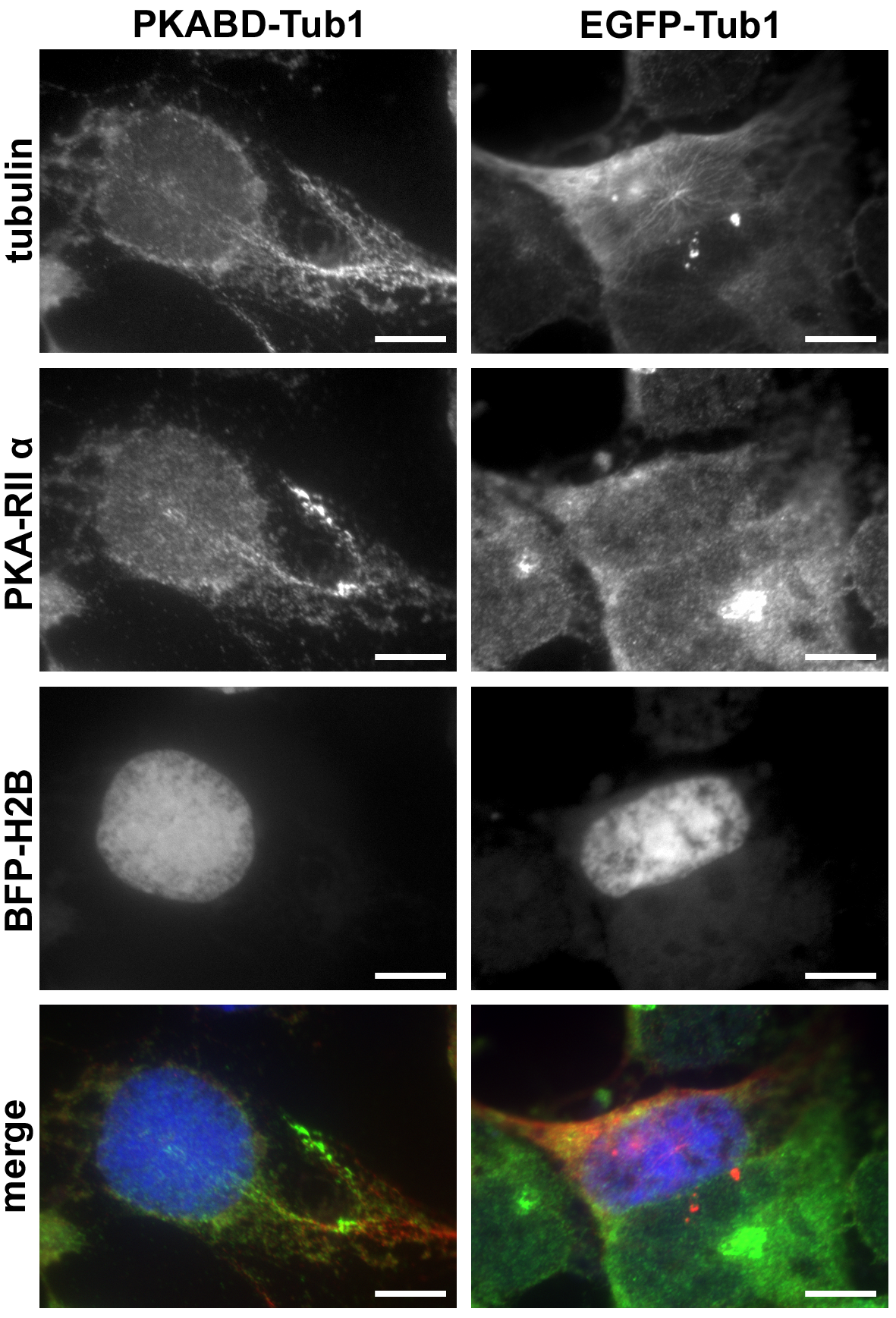

Supplement: Figure S4 — PKA can be recruited onto microtubules in non-neuronal cells by overexpressing the PKABD-Tub1 construct. Images of P19 cells transfected with plasmid overexpressing PKABD-Tub1 (left) or EGFP-Tub1 (right) along with plasmid expressing TagBFP-histone H2B, incubated for 24 hours, and permeabilized with triton X-100 before formaldehyde fixation. Cells were immunofluorescence stained with antibody against tubulin (top row) and PKA-RIIβ (second row). TagBFP-H2B (third row) was used to identify the transfected cells. All scale bars represent 10 µm. (TIF) [file pone.0073890.s004.tif]

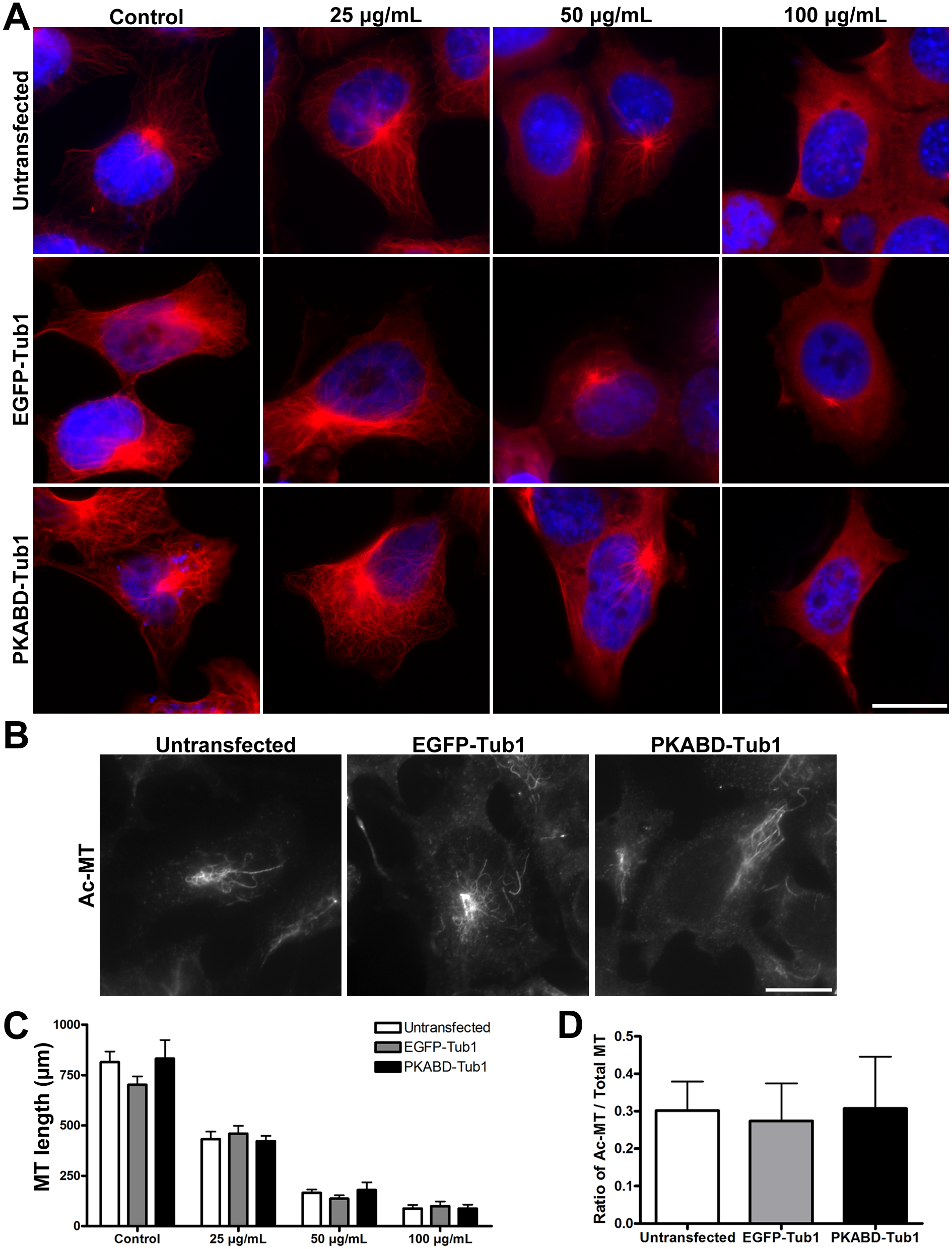

Supplement: Figure S5 — PKABD-Tub1 and EGFP-Tub1 did not affect the stability of the microtubule cytoskeleton. (A) Fluorescent images of untransfected P19 cells (top), P19 cells cotransfected with TagBFP-histone H2B and EGFP-Tub1 (middle), or P19 cells cotransfected with TagBFP-histone H2B and PKABD-Tub1 (bottom) for 23 hours, incubated with 0.2% DMSO (control) or different concentration of nocodazole for 1 hours, and formaldehyde fixed. The α-tubulin staining is shown in red, DAPI staining (top) or TagBFP (middle and bottom) are shown in blue. (B) Fluorescent images of acetylated-microtubule (Ac-MT) in untransfected P19 cells (left), P19 cells transfected with EGFP-Tub1 (middle), or P19 cells transfected with PKABD-Tub1 (right) and fixed at 24 hours. All scale bars represent 20 µm. (C) Quantification of total microtubule length in P19 cells from panel A. (D) Quantification of the ratio of acetylated-microtubules to total microtubules in P19 cells from panel B. All error bars represent SD. More than 15 cells were analyzed per condition per construct. (TIF) [file pone.0073890.s005.tif]
